# Protein multiple sequence alignment benchmarking through secondary structure prediction

**DOI:** 10.1093/bioinformatics/btw840

**Published:** 2017-01-16

**Authors:** Quan Le, Fabian Sievers, Desmond G Higgins

**Affiliations:** Conway Institute, UCD School of Medicine and Medical Science, University College Dublin, Belfield, Dublin, Dublin 4, Ireland

## Abstract

**Motivation:**

Multiple sequence alignment (MSA) is commonly used to analyze sets of homologous protein or DNA sequences. This has lead to the development of many methods and packages for MSA over the past 30 years. Being able to compare different methods has been problematic and has relied on gold standard benchmark datasets of ‘true’ alignments or on MSA simulations. A number of protein benchmark datasets have been produced which rely on a combination of manual alignment and/or automated superposition of protein structures. These are either restricted to very small MSAs with few sequences or require manual alignment which can be subjective. In both cases, it remains very difficult to properly test MSAs of more than a few dozen sequences. PREFAB and HomFam both rely on using a small subset of sequences of known structure and do not fairly test the quality of a full MSA.

**Results:**

In this paper we describe QuanTest, a fully automated and highly scalable test system for protein MSAs which is based on using secondary structure prediction accuracy (SSPA) to measure alignment quality. This is based on the assumption that better MSAs will give more accurate secondary structure predictions when we include sequences of known structure. SSPA measures the quality of an entire alignment however, not just the accuracy on a handful of selected sequences. It can be scaled to alignments of any size but here we demonstrate its use on alignments of either 200 or 1000 sequences. This allows the testing of slow accurate programs as well as faster, less accurate ones. We show that the scores from QuanTest are highly correlated with existing benchmark scores. We also validate the method by comparing a wide range of MSA alignment options and by including different levels of mis-alignment into MSA, and examining the effects on the scores.

**Availability and Implementation:**

QuanTest is available from http://www.bioinf.ucd.ie/download/QuanTest.tgz

**Supplementary information:**

Supplementary data are available at *Bioinformatics* online.

## 1 Introduction

Multiple sequence alignment (MSA) is a very common first step in the analysis of sets of homologous protein sequences (Chatzou *et al.*, 2015) and is an essential precursor to most phylogenetic analyses. In protein structure prediction, the use of MSAs as input for the predictor is the single most important step that helps to improve the prediction accuracy (Cuff and Barton, 2000; Jones *et al.*, 2012; Marks *et al.*, 2012). Recent developments in genome sequencing push further the need for reliable and fast MSA methods. This applies both to the number of alignments, and the size of each alignment.

Despite the widespread use of MSA, all widely used MSA algorithms remain largely heuristic due to the complexity of the optimization algorithms and the difficulty in determining which objective function to optimize (Chatzou *et al.*, 2015). This leads to a critical need for reliable MSA benchmarks (Iantorno *et al.*, 2014) to compare the quality and scalability between different heuristic solutions.

The first large scale protein sequence benchmark for testing MSA algorithms was BAliBASE (Thompson *et al.*, 1999a). This is a manually curated set of alignments, based on protein structure information which assembles sets of test alignments, representing different types of alignment situations. It is widely used and has been very useful in MSA methods development but is constrained by the relatively small sizes of most test alignments and the need for manual intervention (Edgar, 2010). Since then, several other structure based benchmarks have been produced, employing varying amounts of automation (Edgar, 2004; Raghava *et al.*, 2003). All of these suffer from a lack in coverage of some protein types and are also not straightforward to apply to testing large alignments of say thousands of sequences. Simulation based benchmarks (Tan *et al.*, 2015) depend on how well the underlying model reflects reality (Boyce *et al.*, 2015). Embedded benchmarks like HomFam (Blackshields *et al.*, 2010) are comprised of arbitrarily large numbers of sequences but only measure the alignment quality of a tiny number of embedded sequences. Phylogenetic benchmarks (Dessimoz and Gil, 2010) rely on phylogenetic reconstruction methods.

Recently, ContTest, a benchmark method based on contact map prediction was described in (Fox *et al.*, 2015). It is based on measuring the quality of alignments comprised of large numbers of sequences, using information from all sequences and all columns in the alignment. This method uses co-evolution of alignment columns to predict the contact map of real protein sequences, for which accurate 3D structural information is known. Prediction quality in ContTest depends on the ratio of number of sequences in the alignment to the length of the proteins. ContTest will therefore not work well for alignments of fewer than 1000 sequences.

In this paper, we describe an approach to MSA benchmarking which we call QuanTest, based on protein secondary structure prediction. This uses automated predictions of the three main local protein secondary structural types (alpha helix, beta strand, and coil) each amino acid of a protein sequence belongs to (Jones, 1999; Drozdetskiy *et al.*, 2015; Pollastri and McLysaght, 2005). Since the use of MSA helps to improve protein secondary structure prediction accuracy (SSPA), our benchmark is based on the assumption that the better the SSPA, the better the MSA. The sequence data we use to build our benchmark are an extension of the HomFam dataset. We use the widely used and very successful JPred program (Drozdetskiy *et al.*, 2015) as the secondary structure prediction tool. We also construct our benchmark in such a way that we are able to compare our prediction accuracy score with the most widely used existing quality measure available on our benchmark, namely the Sum-of-Pairs score (SPS) (Thompson *et al.*, 1999b).

We set up two sets of test sequences, one with a small number of sequences (200) in each test case to test low throughput accurate aligners, and the second with more sequences in each test case (1000) to test high throughput aligners. We validate the method by comparing the results with SPS scores from structural alignment comparisons and by creating alignments with increasing amounts of random alignment error.

## 2 Materials and methods

### 2.1 Dataset

To build the dataset for our benchmark we first choose all homologous Pfam family—Homstrad pairs where each Homstrad family has at least three reference sequences. We furthermore filter all duplicate sequences from all the Pfam families using the Cd-hit program (Li and Godzik, 2006).

There are two benchmark configurations in our experiments. In the scalability configuration, we choose all Pfam family + Homstrad pairs with more than 6000 unique sequences, resulting in 151 Pfam + Homstrad pairs. In the accuracy benchmark, we choose all Pfam family + Homstrad pairs with more than 1000 unique sequences, resulting in 238 Pfam—Homstrad pairs.

The next step is to generate the test sets of sequences of a chosen size *n_c_*. For each Pfam + Homstrad pair firstly all *n_h_* available Homstrad sequences are selected. Three of these sequences are randomly assigned as reference sequences. Next, $n_{c}-n_{h}$ Pfam sequences are added to the *n_h_* Homstrad sequences to make up test sets of *n_c_* sequences. For both, the scalability and the accuracy configurations, this last step is repeated five times, however, the selection of Homstrad sequences and the assignment of references is kept constant across the resamplings, and across the different *n_c_*. In the scalability configuration initial test sets of *n_c_* = 4000 sequences are generated. Smaller test sets with $n_{c}<4000$ sequences are formed by taking the first *n_c_* sequences from the initial test sets of 4000 sequences, such that larger test sets are always super-sets of smaller test sets. For the accuracy configuration sequences are sampled independently, such that non-reference sequences from the *n_c_* = 200 test sets might not be included in the *n_c_* = 1000 test sets.

We report here some statistics for the accuracy benchmark of 238 Pfam + Homstrad pairs. For the reference alignments, their lengths go from 39 to 887. The average sequence identity between the reference secondary structure sequences in the reference alignments go from 59.73% to 98.54%. The average sequence identities of the reference alignments go from 9.3% to 86.7%, with the median of 33.66%. For the 200 sequence test cases, the average lengths of the Pfam sequences go from 23.5 to 548.7.

### 2.2 Benchmark protocol

We use each aligner setting to align all the test sequence sets in the benchmark (200 sequence setting or 1000 sequence settings). For each chosen reference sequence from an alignment, the program generates its corresponding filtered alignment by deleting all columns from the alignment which correspond to a gap in the reference sequence. Three filtered alignments are generated for each alignment, corresponding to three chosen reference sequences. We then submit all the filtered alignments to the Jpred server, using its RESTful API (v.1.5), to predict the secondary structure of their corresponding reference sequences. For each predicted secondary structure sequence, we calculate its confusion matrix—the 3 × 3 contingency table calibrating the reference secondary structure states (alpha-helix, beta-strand, coil) versus the predicted states—by comparing it with the reference secondary structure extracted from the Homstrad database. The accuracy of the secondary structure prediction for one reference sequence of each filtered alignment is calculated by summing the diagonal elements of the confusion matrix and dividing it by the sum of all of the elements of the confusion matrix.

To make a fair comparison, we only keep the sets of Pfam family + reference sequence + sample index where the filtered alignments of all aligner settings generate a predicted secondary structure from the Jpred server.

The program then calculates the average prediction accuracy of one aligner setting as the average prediction accuracy of all of its chosen filtered alignments.

### 2.3 Aligners

The programs we use to test QuanTest are:

 T-Coffee Version 11.00.8cbe486 (Notredame *et al.*, 2000). We use T-Coffee in the default mode with the command:

t_coffee -output = fasta_aln -n_core = 1 -quiet -no_warning -in = in_file -outfile = out_file

Clustal W2 Version 2.1 (Larkin *et al.*, 2007). We use Clustal W2 in the default mode with the command:

clustalw2 -quiet -output = fasta -n_core = 1 -infile = in_file -outfile = out_file

Clustal Omega Version 1.2.0−r289 (Sievers *et al.*, 2011). We run Clustal Omega in 3 modes. The default mode uses the parameters:

clustalo –outfmt = vie -i in_file -o out_file

While the iteration mode of Clustal Omega uses the parameters:clustalo –iter = num_iter –outfmt = vie -i in_file -o out_file

MUSCLE Version v3.8.31 (Edgar, 2004), to run Muscle for a specified num_iter of iteration:

muscle -quiet -maxiters num_iter -in in_file -out out_file

MUSCLE fast mode with 1 iteration:muscle -quiet -maxiters 1 -diags1 -sv -distance kbit20_3 -in in_file -out out_file

MUSCLE default mode:

muscle -in in_file -out out_file

MAFFT Version v7.245 (Katoh and Standley, 2013). The program use MAFFT in 4 modes. MAFFT default mode:

mafft –quiet –anysymbol in_file > out_file

MAFFT fast mode:

mafft –quiet –retree 1 –maxiterate 0 –nofft –parttree –anysymbol in_file > out_file

MAFFT L-INS-i - Mafft in consistency mode:

linsi –quiet –anysymbol in_file > out_file

MAFFT L-INS-1 - the progressive counterpart of MAFFT L-INS-i:

linsi –maxiterate 0 –quiet –anysymbol in_file > out_file

Kalign 2 version 2.04 (Lassmann and Sonnhammer, 2005) in default mode:

kalign -format fasta -quiet -i in_file -o outfile

Hmmalign from HMMER version 3.1b2 (Eddy, 1998) in default mode:

hmmalign -outformat Stockholm -o out_file hmm_file in_file

Pasta (Mirarab *et al.*, 2014) in default mode:

run_pasta.py -d Protein -i input_file -o output_dir

### 2.4 Correlation between the prediction accuracy and the SPS score

For each Pfam + Homstrad pair, we build a three sequence reference alignment by extracting the lines corresponding to the three reference Homstrad sequences from the Homstrad alignment.

For each predicted secondary structure sequence, we use its corresponding unfiltered alignment to compute its SPS score against the reference alignment, using qscore (Edgar, 2004). We use all residues in the reference alignments to calculate the SPS scores.

## 3 Results

### 3.1 The datasets

The core of our benchmark system is a set of test cases, each of which consists of a large collection of homologous sequences from a family of Pfam 28.0 (Finn *et al.*, 2013) which contains at least three reference sequences of known 3D structure. The reference sequences are taken from a family of the Homstrad alignment database (Mizuguchi *et al.*, 1998). This is similar to how the HomFam (Blackshields *et al.*, 2010) test system is generated and the datasets used here are effectively an extension of HomFam. The basis of all our testing is then to take each of these test cases and make a multiple alignment, using a standard multiple alignment program. Then we predict the secondary structure of each test alignment using JPred4 (Drozdetskiy *et al.*, 2015). This predicted secondary structure is compared to the true secondary structures as seen in the Homstrad references and the overall score is the percentage of positions that are correctly predicted.

**Fig. 1:**
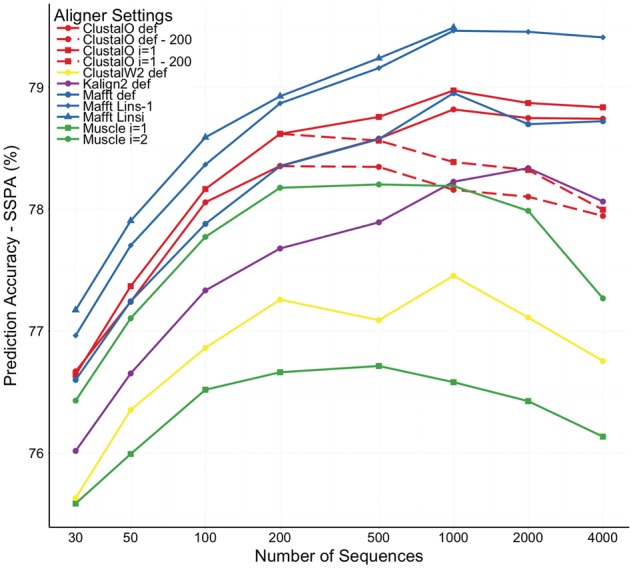
Graph of average prediction accuracy versus number of sequences in alignments for 151 families—solid lines for full alignments of different aligner settings, and dashed lines for sub alignments of 200 sequences embedded in full alignments

For initial testing, we wished to get an idea as to how alignment accuracy varies with different sizes of test cases (different numbers of sequences). For this scalability configuration, we used a test set of Pfam + Homstrad pairs, with at least 6000 unique sequences. 151 families satisfy this criterion. Each test case was resampled to produce smaller test cases of between 30 and 4000 sequences. At each test case size, the subsets were resampled 5 times. This produced 755 alignments to be tested and the results are shown in Figure 1 and discussed in the next section. The details of all of these test cases are given in the supplemental information.

For the secondary structure prediction accuracy (SSPA) benchmark itself, we generated test cases of either 200 or 1000 sequences, sampled from the bigger datasets. This allowed us to compare two broad categories of alignment program: high accuracy and low capacity or lower accuracy and higher capacity. Both have 238 test cases, randomly sampled five times from the full Pfam families, to give a total of 1190 test alignments in each case. Each test alignment consists of 197 non-reference and 3 Homstrad reference sequences or 997 non-reference and three Homstrad reference sequences.

### 3.2 The effect of number of sequences in the alignment

We first tested how the secondary structure prediction accuracy depends on the number of sequences in the alignments. We used our first scalability configuration of 151 Pfam families, each with at least 6000 unique sequences (the data is available at http://www.bioinf.ucd.ie/download/QuanTestScl.tgz). We chose subsets of different numbers of sequences in the alignments according to a log scale: 30,50,100,200,500,1000,2000,4000 sequences. The aligner settings to build the alignments included: Clustal Omega in default mode (Sievers *et al.*, 2011), Clustal Omega with 1 iteration, Clustal W2 in default mode (Larkin *et al.*, 2007), MUSCLE with 1 iteration (Edgar, 2004), MUSCLE with 2 iterations—suggested setting by MUSCLE guideline when aligning more than 1000 sequences, MAFFT in default mode (Katoh and Standley, 2013), MAFFT L-INS-1, and MAFFT L-INS-i, and Kalign 2 in default mode (Lassmann and Sonnhammer, 2005). We included two extra sets of data points for Clustal Omega default and Clustal Omega with 1 iteration. These show the QuanTest SSPA score for alignments of $n_{c}\geq 200$ sequences but where the SSPA is only based on a sub set of 200 sequences.

Figure 1 shows that for the alignments from most aligners, the prediction accuracy improves with the number of sequences up to 1000. After that the accuracy tends to decrease at a slow rate. For Kalign2, the prediction accuracy peaks at 2000 sequences before decreasing at 4000 sequences. For MUSCLE, the prediction accuracy of the alignments reaches its peak at 200 sequences. It then plateaus from 200 to 1000 sequences, and decreases after that.

For the same aligner, Figure 1 shows that options with iterative refinement step always perform better than the progressive counterparts.

For both Clustal Omega options, the SSPA score of the subalignments of 200 sequences embedded in bigger alignments decreases when the total number of sequences to be aligned increases.

In principle, we could continue testing with bigger numbers of sequences to see the full prediction accuracy curve. However, there are not enough Pfam families with sufficiently large numbers of unique sequences (e.g. 10 000 non-reference sequences) for us to get stable and significant results.

### 3.3 Effect of reference and non-reference sequence selection

The prediction accuracy could depend on the selection of reference and/or non-reference sequences. A reference sequence in this context is a sequence for which the true secondary structure is known and is going to be predicted. For this analysis we always selected three Homstrad reference sequences per family. A non-reference sequence is either a Pfam sequence for which no reliable secondary structure is known or a Homstrad sequence whose secondary structure is not to be predicted. For 238 families, we selected 197 non-reference sequences, resampled five times from the larger datasets. The reference sequences were kept fixed for each re-sample of non-reference sequences. All alignments were therefore comprised of 200 sequences. This gave 5 × 238 = 1190 alignments and 3 × 238 = 714 reference sequences. For each of the 1190 sets of sequences, we generated a MSA using Clustal Omega in default mode.

**Fig. 2:**
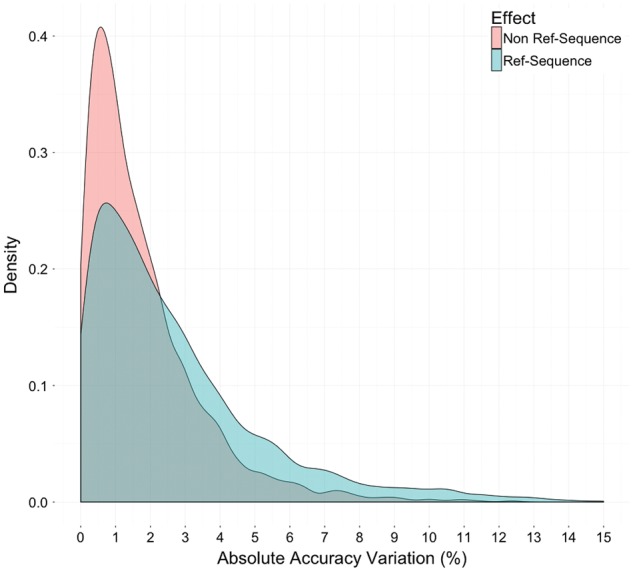
The variation of prediction accuracy according to the choice of three reference sequences and the non-reference sequences in five samples. In blue is the variation of prediction accuracy among three reference sequences in the same alignment; in red is the variation of prediction accuracy of the same reference sequence among five samples of the same Pfam family (Color version of this figure is available at *Bioinformatics* online.)

For each of the 1190 alignments we calculated the average prediction accuracy over the three reference sequences. To quantify the effect of reference sequence selection we determined, for each sequence, the absolute difference of its prediction accuracy from the average of three. The distribution of these differences is shown in Figure 2 in blue.

Next we calculated for each of the 714 reference sequences the average prediction accuracy over the five re-samples. To quantify the effect of non-reference sequence selection we determined, for each sequence, the absolute difference of its prediction accuracy from the average over the re-samples. This distribution is depicted in Figure 2 in red.

One can see in Figure 2 that reference sequence selection has a bigger effect than non-reference sequence selection. The mean absolute prediction accuracy variation due to reference sequence selection is 2.74% (blue). This is much bigger than the mean absolute prediction accuracy variation due to non-reference sequence selection of 1.77% (red). The difference between the means of two score distributions is significant with *P*-value of 0.0005 according to the Mann-Whitney U test. For this reason we did not rely on just one reference sequence but take the average over three reference sequences for the subsequent analyses.

### 3.4 Correlation between SSPA and SPS score

**Fig. 3:**
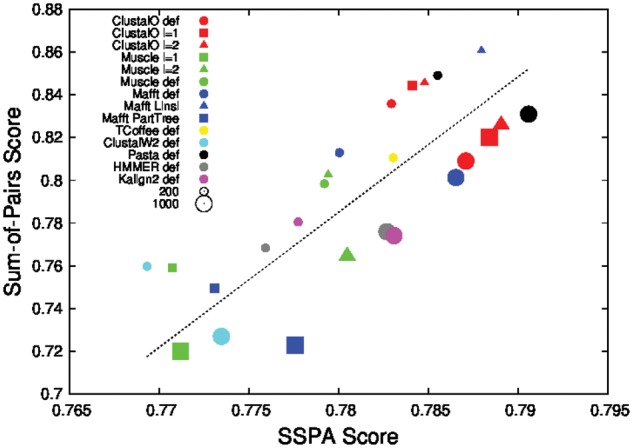
Average Prediction Accuracy versus Average SPS Score for alignments of 200 and 1000 sequences from 238 Pfam families

We compared the prediction accuracy of the alignments with the traditional alignment quality score: the Sum-of-Pairs score (SPS score) (Thompson *et al.*, 1999b). Figure 3 plots the average SPS scores against the average prediction accuracy (SSPA) for the 200 and 1000 sequence alignments on the upper left, and lower right, respectively. The lower-right section of Figure 3 includes fewer data points because high quality, low throughput aligners were omitted for these comparisons.

For both numbers of sequences, Figure 3 shows almost linear correlation between the prediction accuracy and the SPS scores, although the average SPS scores are spread over a bigger range than the average prediction accuracy. For alignments of 200 sequences the average SPS score range is 75.8–86.7, while the prediction accuracy range is 77.1–78.9%.

We also observe that increasing the number of sequences increases the SSPA. This is consistent with the results in Figure 1. On the other hand increasing the number of sequences in the alignments decreases the SPS score, consistent with previous results (Sievers *et al.*, 2013) and the dashed lines in Figure 1. For Clustal Omega in default mode, going from 200 sequences to 1000 sequences improves the prediction accuracy from 78.45% to 78.73%, while it decreases the SPS score from 84.22% to 81.0%. One exception is the result for alignments built by hmmalign of HMMER. Since hmmalign builds an alignment by aligning individual sequences against the Pfam profile HMM, the average SPS score stays constant at 77.9% when going from 200 sequences to 1000 sequences while its SSPA score goes up from 77.86% to 78.32%

### 3.5 Effect of adding errors to the alignments

We tested how adding errors to the alignments changes the prediction accuracy. We followed the same protocol as in (Fox *et al.*, 2015), where a fixed percentage of aligned sequences were shifted to the right by one position. We used the alignments built by Clustal Omega in default mode from the accuracy benchmark configuration of 200 sequences. Then 5%,10%,15% of sequences were shifted. For each error level we repeated the process 10 times to measure the variation in prediction accuracy.

**Fig. 4:**
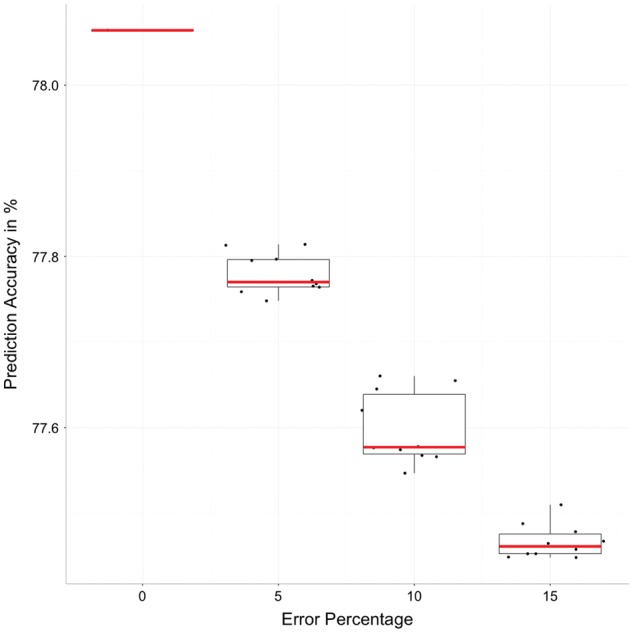
Effect on prediction accuracy when adding errors to Clustal Omega alignments of 200 sequences on the SSPA. The boxplot for each percentage of error is created from 10 resamples: the whiskers represent the top and bottom 25% quantiles, the red line is the median

The results in Figure 4 show strong negative correlation between alignment error added and the prediction accuracy. This is consistent with our assumption that good alignments lead to good SSPA and conversely that alignment errors degrade the SSPA.

### 3.6 Benchmarking aligners for 200 sequences

This experiment used the accuracy configuration of 238 Pfam families, each with 5 samples of 200 sequences. We benchmarked the alignments of a wide range of aligners and options: Clustal W2 in default mode (Larkin *et al.*, 2007), Clustal Omega in default mode, Clustal Omega with 1 iteration, Clustal Omega with 2 iterations, MUSCLE with 1 iteration, MUSCLE with 2 iterations, MUSCLE in default mode with 16 iterations, MAFFT fast mode without reestimating the guide tree, MAFFT in default mode, Kalign 2, and two consistency based aligners: MAFFT in consistency mode (MAFFT L-INS-i), T-Coffee (Notredame *et al.*, 2000) in default mode, HMMER in default mode (Eddy, 1998), and PASTA in default mode (Mirarab *et al.*, 2014).

Table 1 shows the results of different aligner settings. The ranking largely agrees with our expectation where for the same aligner the alignments from ‘higher accuracy’ settings with more refinement lead to better prediction accuracy. The differences are small but they are significant at *p* < 0.01. T-Coffee performs on a par with Clustal Omega in default mode, while the difference in average SSPA scores between MUSCLE—2 iterations and MUSCLE—default mode with 16 iterations is not significant.

**Table 1:** The prediction accuracy for alignments of 200 sequences for 238 Pfam families

Aligner settings	Prediction Accuracy (in %)
MAFFT L-INS-i	78.94 *
MAFFT—Default	78.19
MAFFT—Fast Mode	77.53 *
Clustal Omega—2 iter	78.36 *
Clustal Omega—1 iter	78.56 *
Clustal Omega—Default	78.63
MUSCLE—2 iter	78.17
MUSCLE—Default	78.13
MUSCLE—1 iter	77.29 *
PASTA—Default	78.70
T-Coffee—Default	78.45
Kalign 2—Default	77.93
Clustal W2—Default	77.13
HMMER—Default	77.86

For aligner settings from the same aligner, the sign (*) signifies that the score is significantly different (higher or lower) from the default score with *P* < 0.01 using the Wilcoxon signed rank test.

### 3.7 Benchmarking aligners for 1000 sequences

Here we used the accuracy configuration of 238 Pfam families, with 5 samples of 1000 sequences for each Pfam family. The experiment considered only aligner options which can build an alignment from a bigger number of sequences in reasonable time: Clustal W2 in default mode, Clustal Omega in default mode, Clustal Omega with 1 iteration, Clustal Omega with 2 iterations, MUSCLE with 1 iteration, MUSCLE with 2 iterations, MAFFT fast mode, MAFFT in default mode, Kalign 2, HMMER in default mode, and PASTA in default mode.

Figure 3 compares the prediction accuracy of the alignments of 200 sequences versus 1000 sequences for different aligner settings. The ranking between different aligner settings stays largely the same: the best group includes PASTA, Clustal Omega with 1 iteration and Clustal Omega with two iterations. The second group includes Clustal Omega—default mode, MAFFT—default mode; the third group includes Kalign 2, HMMER, and MUSCLE-2 iterations. The fourth group includes MAFFT—Fast Mode; Clustal W2—default mode and MUSCLE 1 iteration are in the final group. The improvement in prediction accuracy is noticeable when going from alignments of 200 sequences to alignments of 1000 sequences—except for the MUSCLE aligner settings whose prediction accuracies stay almost the same: the accuracy of Clustal Omega alignments improves from 78.45% to 78.73%, with similar improvements for other aligner settings.

## 4 Conclusion

QuanTest is based on using SSPA as a proxy for multiple sequence alignment accuracy or quality. It behaves well as benchmark for several reasons. First it correlates well with the main existing measure of alignment quality: SPS score of a small set of reference sequences against a structure based alignment of the same sequences. Secondly, when we add alignment errors in a controlled fashion, increasing levels of error cause a monotonic and regular decrease in QuanTest score. Finally, when we compare different alignment options of various programs, we mainly see the ‘higher accuracy’ options, giving better scores. This latter point is potentially circular but we do see that the use of iterations or the use of consistency, for example, giving higher scores.

The question then is, if QuanTest gives basically the same answer as SPS score, why use QuanTest? QuanTest is much more easily automated to give benchmark test sets of almost any size and for any subtype of proteins, as long as one or more sequences have known secondary structure. In all of the cases here, this is based on using 3D structures to infer secondary structure. This process is much easier to automate completely reliably, than multiple structure superposition (Krissinel and Henrick, 2005; Kolodny *et al.*, 2005). Several widely used and highly effective algorithms exist for the latter (Taylor *et al.*, 1994; Konagurthu *et al.*, 2006; Guda *et al.*, 2004) so it is possible to do but the algorithms are heuristic (Hasegawa and Holm, 2009) and there are differences between different approaches for doing so.

A further reason for using QuanTest is that it is based on all of the sequences in an alignment. SPS scores are based only on the subset of sequences of known structure. This subset can be tiny and unrepresentative so that testing very large alignments can be problematic (Blackshields *et al.*, 2010). The reference sequences can cluster together and are aligned against one another first. In such a case the quality of the subalignment of the reference sequences does not reflect the quality of the whole alignment. With QuanTest, all of the sequences can contribute to the score and this gives a more realistic test of big alignments.

Compared to the ContTest score, the QuanTest SSPA score is less dependent on the lengths of the reference protein sequences, and it can be used for alignments comprised of fewer sequences than ContTest. In this study we demonstrate the usefulness of QuanTest for 200 sequences, whereas ContTest would typically require more than 1000 sequences to give an accurate scoring.

Clearly, the method presented here will only work for protein sequences. A further restriction is that it will not work for sequences whose 3D structure is hard to determine. It will be heavily biased towards single domain globular proteins, but this bias is true of most structure based protein benchmarks. BALiBASE is a notable exception, in that it does include representative alignments from outside of this range but it is hard to scale it to many test cases or very big alignments. In the current test sets, we have been quite restrictive regarding the number and type of test examples. These numbers can easily be expanded to include literally thousands of test cases of almost any size. Other protein structure prediction measures (Taylor, 2016)—such as solvent accessibility accuracy- could be added to the SSPA score to assess the alignment quality from different view points, while the need for true secondary structures of reference sequences could be removed if we replace them by the consensus secondary structures—voted by predicted secondary structures when varying the set of homologous sequences or varying the aligner settings to build the alignment. As such QuanTest’s protocol represents a powerful and flexible way to test MSA alignment quality where the alignments are being used to detect or demonstrate homology or for structure prediction. It remains an open question as to how well a benchmark such as this one will reflect the usefulness of methods for phylogenetic reconstruction (Iantorno *et al.*, 2014).

## Supplementary Material

Supplementary Data
