# Supplementary material for: Protein multiple sequence alignment benchmarking through secondary structure prediction
Source: Bioinformatics. 2017 Jan 16;33(9):1331–7. doi: 10.1093/bioinformatics/btw840 (PMC5408826; doi:10.1093/bioinformatics/btw840)
Supplement: Supplementary Data [file btw840_supp.pdf]

Supplementary:Protein Multiple Sequence Alignment Benchmarking through Secondary Structure Prediction

December 9, 2016

Pfam - Homstrad Families in the Benchmark

Table S1: Statistics of the 238 Pfam-Homstrad pairs in the Benchmark.

Pfam Id	Homstrad Id	N Uniq Seqs (Pfam)	Avg Length (Pfam 200 seq samples)	Avg Length (Homstrad ref. seqs)	Avg SS Id (Homstrad ref. seqs)
PF00004	AAA	28280	134.7	336.7	72.75%
PF00497	aabp	7833	221.4	232.3	84.38%
PF00135	ace	13714	415.5	530.7	83.75%
PF00328	acid_phosphat	5371	309.9	372.7	80.21%
PF01648	ACPS	8302	109.4	109.7	75.92%
PF00709	Adenylsucc_synt	6577	377.5	430.7	88.68%
PF00241	ADF	3238	122.2	147.3	75.11%
PF00578	AhpC-TSA	22198	127.4	195.7	86.46%
PF00171	aldedh	20335	412.8	481.0	84.64%
PF00274	aldose	2498	268.1	355.0	92.59%
PF00248	aldosered	16845	263.7	318.0	77.95%
PF02806	alpha-amylase_C	5060	92.9	89.0	75.89%
PF00023	ANK	3320	35.4	127.3	83.77%
PF01261	AP_endonuc_2	20433	211.0	271.0	76.66%
PF00514	ARM	10569	46.8	431.0	77.68%
PF00026	asp	7465	291.2	336.7	79.32%
PF01177	Asp_Glu_race_D	9493	205.9	111.3	61.49%
PF00127	az	2610	104.0	109.3	73.80%
PF00216	Bac_DNA_binding	9015	88.4	88.7	83.24%
PF00296	bac_luciferase	24615	296.9	292.3	77.75%
PF01036	Bac_rhodopsin	3498	150.4	226.0	89.03%
PF01011	Bacterial_PQQ	1947	44.1	582.7	89.01%
PF00373	Band_41_M	3755	134.7	109.0	89.65%
PF00373	Band_41_N	3755	134.4	83.7	82.30%
PF00364	biotin_lipoyl	22567	71.7	80.0	68.75%
PF00653	BIR	2049	69.5	114.0	71.70%
PF01453	B_lectin	4587	98.9	108.7	96.62%
PF00753	blmb	41391	185.4	247.3	77.92%
PF00439	bromodomain	8223	84.9	117.3	91.53%
PF00130	C1	4056	52.6	56.0	76.33%
PF00168	C2	27465	105.6	129.7	82.06%
PF00194	cah	4352	216.0	256.0	87.19%
PF00199	cat	7183	342.2	566.7	93.21%
PF02839	cbm12	2479	42.1	52.0	73.18%
PF00686	CBM_20	1808	93.8	103.3	87.30%
PF00571	CBS	65876	57.4	59.0	76.37%
PF00084	ccH	19433	56.3	59.3	75.53%
PF00028	cdh	38924	93.5	104.3	84.56%
PF00307	CH	10001	103.4	115.7	81.48%

Table S1 – continued from previous page

Pfam Id	Homstrad Id	N Uniq Seqs (Pfam)	Avg Length (Pfam 200 seq samples)	Avg Length (Homstrad ref. seqs)	Avg SS Id (Homstrad ref. seqs)
PF00187	ChtBD	1398	40.9	42.7	85.83%
PF00285	citrate_synt	9993	295.1	394.3	87.23%
PF00510	COX3	14347	217.7	239.7	76.57%
PF00246	cpa	7738	246.6	312.3	92.07%
PF02786	CPS	25494	197.1	240.7	86.29%
PF00313	csp	10707	65.0	67.3	91.46%
PF00431	CUB	12533	106.7	110.7	95.43%
PF02298	Cu_bind_like	2211	81.9	99.0	83.84%
PF00394	Cu_nir	12349	147.2	334.0	97.50%
PF00160	cyclo	16006	157.6	173.7	81.90%
PF00112	cys	9834	202.3	240.0	85.55%
PF01053	Cys_Met_Meta_PP	17111	366.9	390.0	88.67%
PF00548	Cys-protease-3C	1740	160.5	191.0	82.59%
PF00031	cystatin	1959	88.9	101.3	64.29%
PF00173	cytb	8094	83.4	84.0	76.20%
PF00034	cytc	12687	98.7	105.3	83.39%
PF00793	DAHP_synth_1	9544	283.8	295.3	70.25%
PF00383	dCMP_cyt_deam	18209	105.6	120.3	82.14%
PF00270	DEAD	32915	174.6	270.7	68.55%
PF00531	DEATH	2641	81.5	127.0	75.95%
PF00186	dhfr	5383	160.5	175.7	81.29%
PF02833	DHHA2	2054	130.4	119.7	83.65%
PF01368	DHH	9183	164.7	185.7	90.22%
PF01180	DHOdehase	8126	276.4	327.7	78.27%
PF00200	DISIN	2233	73.7	62.3	79.75%
PF00885	DMRL_synthase	4351	139.2	160.3	82.35%
PF00035	dsrm	7222	66.3	82.3	79.39%
PF02597	DUF170	7433	73.4	72.3	72.77%
PF00692	dutpase	7623	123.1	124.3	90.98%
PF00378	ech	12309	235.0	266.0	84.41%
PF00736	EF1BD	1093	84.4	90.0	83.57%
PF03144	EFTU_2	25545	70.7	103.3	83.46%
PF03143	EFTU_C	11795	92.1	97.7	90.90%
PF00008	egf	21300	32.7	49.7	78.77%
PF00053	EGF_Lam	15714	49.4	54.0	71.30%
PF01417	ENTH	1275	121.9	225.7	82.82%
PF00178	ets	1217	79.3	94.0	70.01%
PF00903	Extradiol_dioxy	20287	130.4	290.7	88.76%
PF00061	fabp	2688	129.8	133.0	91.09%
PF01565	FAD_binding_4	25883	136.4	238.7	73.54%
PF02913	FAD-oxidase_C	11879	230.7	267.7	77.39%
PF01149	Fapy_DNA_glyco	4691	131.9	261.0	89.23%
PF00316	fbpase	3390	303.1	322.3	82.95%

Table S1 – continued from previous page

Pfam Id	Homstrad Id	N Uniq Seqs (Pfam)	Avg Length (Pfam 200 seq samples)	Avg Length (Homstrad ref. seqs)	Avg SS Id (Homstrad ref. seqs)
PF00111	fer2	19794	77.0	101.3	62.77%
PF00210	ferritin	9855	137.8	164.7	93.38%
PF00147	fibrinogen_C	4895	171.9	234.7	84.24%
PF00630	Filamin	6156	92.5	104.0	74.71%
PF00254	fkbp	16590	98.9	113.7	91.11%
PF00258	flav	9739	137.4	160.0	79.00%
PF00250	Fork_head	3307	86.0	92.3	74.29%
PF01590	GAF	17869	148.4	156.7	75.65%
PF00172	GAL4	19826	38.9	39.7	93.10%
PF00320	GATA	2616	35.6	59.3	79.80%
PF00117	GATase	12140	193.5	209.0	78.47%
PF00331	ghf10	3538	256.0	307.7	93.03%
PF00457	ghf11	1028	163.6	185.3	86.30%
PF00704	ghf18	12423	288.2	282.3	61.85%
PF00232	ghf1	14892	390.7	440.0	92.52%
PF00062	ghf22	1071	115.7	127.0	92.45%
PF00064	ghf34	18268	397.9	389.0	89.01%
PF00150	ghf5	8173	270.3	308.3	68.29%
PF00840	ghf7	4631	199.8	400.0	80.95%
PF00710	glnasn	6074	301.7	325.3	91.61%
PF00042	glob	5658	110.3	141.3	83.45%
PF00704	Glyco_hydro_18	12423	291.8	422.3	76.05%
PF00704	Glyco_hydro_18_D1	12423	290.3	338.3	73.09%
PF00704	Glyco_hydro_18_D2	12423	285.1	74.3	81.62%
PF00703	Glyco_hydro_2	7981	105.9	107.3	59.73%
PF00358	gpr	5555	126.0	154.0	88.57%
PF00025	gtp	25272	203.0	170.0	85.18%
PF00561	Haloperoxidase	14899	221.1	284.0	75.97%
PF00045	hemopexin	4118	47.9	201.7	80.38%
PF00132	hexapep	50148	37.7	160.7	73.74%
PF03129	HGTP_anticodon	18649	92.3	107.3	87.07%
PF00125	histone	7819	106.4	84.7	86.46%
PF00129	hla	17095	147.5	178.0	95.51%
PF00010	HLH	10289	54.7	70.3	65.66%
PF00403	HMA	20591	60.8	70.7	84.64%
PF00505	HMG_box	7023	66.3	78.7	85.03%
PF00046	hom	13416	55.4	67.3	82.94%
PF00104	hormone_rec	6589	183.0	242.3	72.27%
PF00381	hpr	5745	81.0	87.7	92.76%
PF00105	hr	4168	65.8	73.0	89.23%
PF00180	icd	15495	326.9	358.7	89.33%
PF00048	il8	1896	59.0	71.3	80.43%
PF00459	inositol_P	12553	258.2	342.0	74.04%

Table S1 – continued from previous page

Pfam Id	Homstrad Id	N Uniq Seqs (Pfam)	Avg Length (Pfam 200 seq samples)	Avg Length (Homstrad ref. seqs)	Avg SS Id (Homstrad ref. seqs)
PF00092	int	11426	166.3	197.7	77.92%
PF02369	Invasin	2751	95.8	97.7	82.00%
PF00050	kazal	1898	51.8	56.0	89.29%
PF00450	kex	7032	343.1	437.3	77.81%
PF00225	KISc	13541	296.0	328.0	81.84%
PF00051	kringle	2512	76.8	85.7	87.73%
PF00014	kunitz	6169	53.5	58.3	91.86%
PF00356	lacI	19559	46.0	57.0	75.47%
PF02264	lamb	1583	379.4	418.3	82.70%
PF00054	laminin_G	1489	130.5	184.7	76.14%
PF00057	LDLa	17146	38.2	43.0	81.36%
PF00549	ligase-CoA	7008	124.5	152.7	82.09%
PF00412	LIM	12339	57.5	68.3	77.49%
PF00061	lipocalin	2688	132.0	160.3	82.84%
PF01451	LMWPc	9302	133.1	157.7	95.33%
PF00560	LRR	12825	30.6	419.7	83.64%
PF00677	Lum_binding	8196	85.4	105.0	83.48%
PF02664	LuxS	1910	147.4	148.7	79.59%
PF02249	MCR_alpha	8232	121.3	279.7	95.80%
PF00969	MHC_II_N	2091	76.4	86.3	87.72%
PF02310	MM_CoA_mutase	9697	115.4	143.3	69.59%
PF00413	mmp	3688	163.2	169.7	90.84%
PF00994	MoCF_biosynth	13876	146.3	168.3	79.90%
PF00059	msb	13647	108.8	125.7	76.24%
PF02875	Mur_ligase_C	18978	86.5	127.3	65.66%
PF00249	myb_DNA-binding	16236	47.3	52.7	73.75%
PF02525	NADHdh_2	9517	186.0	258.7	92.93%
PF00334	ndk	5851	131.4	148.7	93.11%
PF00374	NiFeSe_Hases	4471	260.5	535.7	93.97%
PF00881	Nitroreductase	22189	147.7	215.7	77.16%
PF00483	NTP_transferase	27132	236.9	276.3	80.37%
PF00202	oat	7737	375.1	431.7	80.48%
PF00278	Orn_DAP_Arg_deC	9577	135.8	152.3	89.75%
PF00067	p450	29549	344.9	413.3	70.80%
PF00149	PA_phosphatase	20352	211.5	304.7	78.95%
PF00989	PAS	19501	105.7	124.7	76.06%
PF00595	PDZ	17619	80.8	96.0	92.29%
PF00544	pec_lyase	2270	189.5	370.0	62.66%
PF00557	Peptidase_M24	24944	221.5	269.0	74.43%
PF00391	PEP-utilizers	9257	76.8	128.7	62.62%
PF00300	PGAM	11441	158.3	215.0	82.68%
PF00162	pgk	6960	363.0	408.0	87.11%
PF00589	Phage_integrase	10351	168.4	187.3	74.36%

Table S1 – continued from previous page

Pfam Id	Homstrad Id	N Uniq Seqs (Pfam)	Avg Length (Pfam 200 seq samples)	Avg Length (Homstrad ref. seqs)	Avg SS Id (Homstrad ref. seqs)
PF00068	phoslip	1638	108.1	122.0	81.06%
PF00343	phs	6893	548.7	825.7	84.94%
PF00114	pilin	2007	110.2	135.3	60.83%
PF00267	porin	3621	304.7	306.7	77.24%
PF00719	ppase	4077	155.6	210.3	81.46%
PF00484	Pro_CA	5853	150.0	221.0	79.01%
PF00235	profilin	1331	122.4	126.7	90.93%
PF02244	Propep_M14	1173	72.4	92.3	78.52%
PF00156	prt	11756	142.4	173.3	72.76%
PF00640	PTB	2359	128.4	147.0	64.55%
PF00102	ptpase	8041	209.1	284.7	86.11%
PF02796	recombinase	2981	44.5	48.7	86.37%
PF01381	rep	35918	53.9	75.3	86.45%
PF00072	response_reg	70885	113.0	121.7	80.06%
PF00615	RGS	4657	123.9	136.0	77.92%
PF00581	Rhodanese	32677	104.8	146.3	68.30%
PF00687	Ribosomal_L1	6135	197.3	216.3	85.56%
PF00347	Ribosomal_L6_D	9324	75.1	85.7	74.62%
PF01248	Ribosomal_L7Ae	5301	91.3	116.0	83.38%
PF00097	RING	7364	42.8	58.7	67.75%
PF01351	RNase_HII	6819	180.8	210.7	86.55%
PF00074	rnasemam	1014	115.8	119.7	80.38%
PF00075	rnh	15145	126.8	140.0	76.32%
PF00268	rnr	5247	268.9	303.7	84.91%
PF01765	RRF	4687	161.4	184.3	84.78%
PF00076	rrm	55944	69.3	83.7	71.91%
PF00301	rub	2585	46.4	50.3	94.07%
PF00016	RuBisCO_large	36205	258.8	315.0	85.12%
PF02788	RuBisCO_large_N	15020	115.6	130.7	84.32%
PF00077	rvp	106024	94.1	112.0	83.79%
PF01479	S4	24013	46.9	47.7	92.98%
PF02037	SAP	2286	34.5	43.7	84.34%
PF01547	SBP_bacterial_1	12081	291.0	329.0	78.64%
PF00089	serbact	24900	207.6	186.3	92.18%
PF00089	sermam	24900	211.3	245.0	82.51%
PF00079	serpin	7305	312.6	380.3	86.05%
PF00017	sh2	7393	79.1	106.0	80.15%
PF00018	sh3	9550	49.0	67.7	78.54%
PF00337	slectin	2417	133.2	132.0	83.89%
PF01423	Sm	6693	70.0	75.7	78.48%
PF00080	sodcu	4104	137.0	152.3	81.46%
PF00319	SRF-TF	4513	45.8	80.0	91.99%
PF00082	subt	9151	295.5	276.7	98.54%

Table S1 – continued from previous page

Pfam Id	Homstrad Id	N Uniq Seqs (Pfam)	Avg Length (Pfam 200 seq samples)	Avg Length (Homstrad ref. seqs)	Avg SS Id (Homstrad ref. seqs)
PF00532	sugbp	5528	252.2	288.0	85.30%
PF00685	Sulfotransfer	6353	229.8	262.0	66.82%
PF01421	svmp	3158	188.3	199.3	92.08%
PF00352	tbpc	2154	82.9	183.7	96.28%
PF00019	tgfb	2613	96.6	112.0	79.76%
PF00314	THAUMATIN	2087	181.5	207.0	88.52%
PF00462	thiored	27818	101.8	91.3	67.87%
PF01833	TIG	8705	86.3	84.3	93.94%
PF01826	TIL	4285	56.4	67.3	78.56%
PF00121	tim	8515	214.9	249.7	89.20%
PF00303	tms	5329	264.8	288.7	82.75%
PF00229	TNF	1060	122.6	146.3	92.83%
PF01751	Toprim	16697	116.8	110.7	77.02%
PF02780	transketolase_C	18803	116.5	139.0	74.13%
PF02779	transket_pyr	20239	175.0	193.3	68.77%
PF00576	TRANSTHYRETIN	2118	111.8	116.0	90.64%
PF01588	tRNA_bind	9919	99.5	109.3	73.53%
PF00133	tRNA-synt_1	9680	416.7	618.3	74.64%
PF00234	tryp_alpha_amyl	2197	104.6	114.7	75.30%
PF00069	TyrKc	30033	230.5	275.3	84.92%
PF00240	UBQ	9156	66.3	74.3	98.19%
PF00179	uce	12030	133.0	148.7	83.95%
PF03167	ung	11771	160.1	224.3	88.60%
PF01042	UPF0076	13581	115.8	125.3	94.67%
PF00397	WW	5834	30.1	40.7	86.02%
PF02542	YgbB	3943	153.2	153.3	82.79%
PF00096	zf-CCHH	41158	23.5	29.3	74.02%

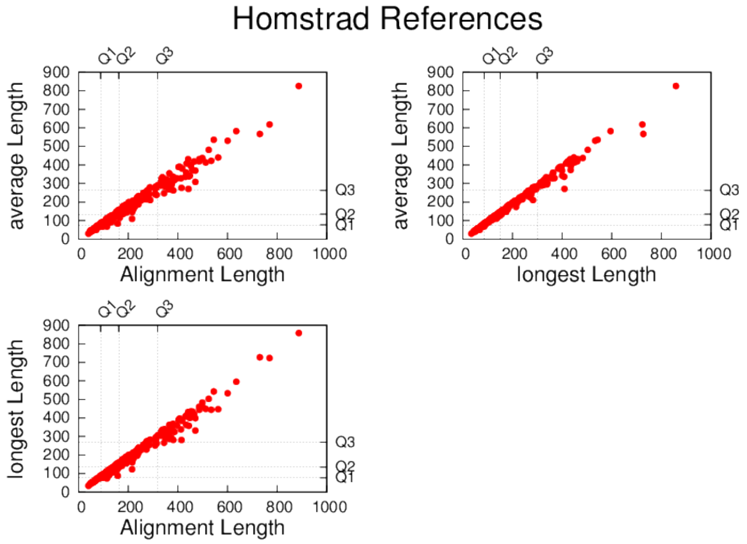

Figure S1: Visualization of the length statistics of the Homstrad references for 238 families. The graph on the top left depicts the length of the reference alignment versus the average length of 3 Homstrad reference sequences for each family ; the graph on the top right depicts the longest length among 3 Homstrad reference sequences for each family versus their average length; the graph on the bottom left depicts the length of the reference alignment versus the longest length of the 3 reference sequences.

Visualizations of the benchmark

Experiment Results

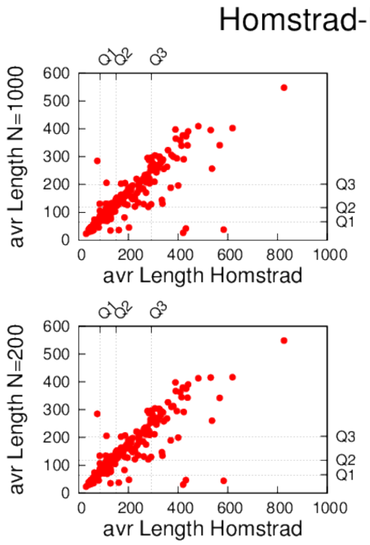

Figure S2: The length statistics of the 238 Homstrad-Pfam pairs in the benchmark. On the top left is the graph of the average length of 1000 sequence samples versus the average length of the 3 Homstrad sequences in the samples; the graph on the top right depicts the average length of the samples of 200 sequences versus the average length of the samples of 1000 sequences. The graph on the bottom left depicts the average length of the Homstrad reference sequences vs the average length of the samples of 200 sequences.

Aligner Settings	Prediction Accuracy (in %)	SP Score (in %)	Total Column Score (in %)
Mafft - L-INS-i	78.94	86.71	81.30
Pasta - Default	78.70	85.56	79.63
Clustal Omega - 2 iter	78.63	85.11	79.54
Clustal Omega - 1 iter	78.56	84.99	79.40
Tcoffee - Default	78.45	81.79	75.11
Clustal O - Default	78.45	84.22	78.33
Mafft - Default	78.20	81.98	75.34
Muscle - 2 iter	78.17	81.20	74.46
Muscle - Default	78.13	80.83	74.33
Kalign 2 - Default	77.93	78.64	71.22
HMMER - Default	77.86	77.91	70.31
Mafft - Fast Mode	77.53	75.78	67.78
Muscle - 1 iter	77.29	76.54	69.05
Clustalw2 - Default	77.13	77.01	69.39

Table S2: Results for the benchmark of 200 sequences for 238 Pfam families

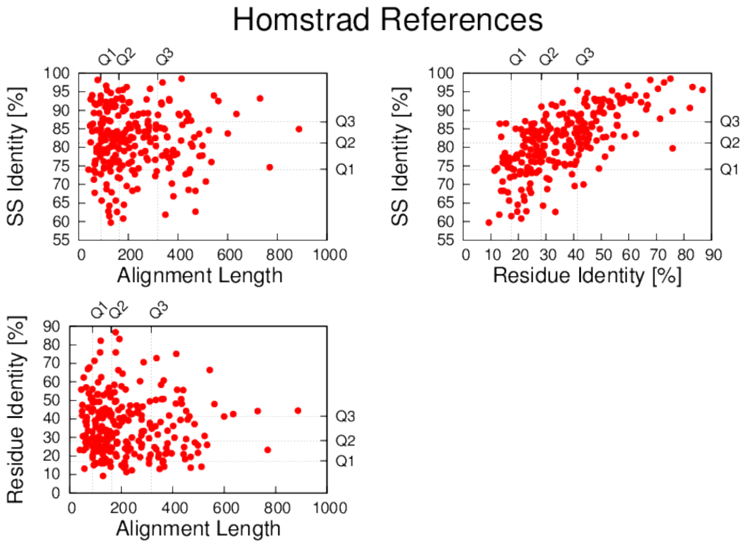

Figure S3: Visualizations of the Homstrad reference alignment statistics. The graph on the top left depicts the alignment length versus the average secondary structure identities; the graph on the top right depicts the average residue identities between Homstrad reference sequences vs the average secondary structure identities between Homstrad reference sequences for each family; the graph on the bottom left depicts the alignment length versus the average residue identities between Homstrad reference sequences for each family.

Aligner Settings	Prediction Accuracy (in %)	SP Score (in %)	Total Column Score (in %)
Pasta - Default	79.10	83.19	76.71
Clustal Omega - 2 iter	78.94	82.69	76.42
Clustal Omega - 1 iter	78.80	82.13	75.79
Clustal Omega - Default	78.73	80.98	74.26
Mafft - Default	78.68	80.36	73.41
Kalign 2 - Default	78.34	77.53	69.77
HMMER - Default	78.31	77.91	70.31
Muscle - 2 iter	78.09	76.72	68.97
Mafft - Fast Mode	77.80	72.51	63.72
Clustalw2 - Default	77.38	72.87	64.06
Muscle - 1 iter	77.15	72.03	63.57

Table S3: Results for the benchmark of 1000 sequences for 238 Pfam families
